# Acquisition, Divergence, and Personalization of the Female Perineal Microbiomes Are Driven by Developmental Milestones and Disrupted by Urinary Tract Infection: A Pilot Study

**DOI:** 10.3389/fped.2020.542413

**Published:** 2020-12-08

**Authors:** Elizabeth J. Lucas, Christina B. Ching, Shweta Saraswat, Shareef M. Dabdoub, Purnima P. Kumar, Sheryl S. Justice

**Affiliations:** ^1^Division of Complex Healthcare, Pediatrics Department, Nationwide Children's Hospital, Columbus, OH, United States; ^2^Division of Pediatric Urology, Nationwide Children's Hospital, Columbus, OH, United States; ^3^Center for Clinical and Translational Research, Research Institute at Nationwide Children's Hospital, Columbus, OH, United States; ^4^College of Dentistry, The Ohio State University, Columbus, OH, United States; ^5^Center for Microbial Pathogenesis, Research Institute at Nationwide Children's Hospital, Columbus, OH, United States

**Keywords:** microbiome, female, child, perineum, urinary tract infections

## Abstract

**Introduction:** The pediatric perineal microbiomes inhabit a dynamic environment with changes related to diet, toileting habits, and hormonal development. We hypothesized that next-generation sequencing would reveal different perineal bacterial signatures associated with developmental milestones in premenstrual females. Furthermore, we predicted that these microbial changes would be disrupted in premenstrual females with a history of urinary tract infection (UTI).

**Study Design:** Healthy females were recruited at well-child visits. Subjects were divided into 4 developmental groups: (1) 0–3 month old newborns; (2) 4–10 month old infants transitioning to solid foods; (3) 2–6 year old toddlers peri-toilet training; and (4) 7–12 year old premenstrual girls. A separate group of females with a history of culture proven UTI and off antibiotics >1 month was also recruited. DNA was isolated from swabs of the perineum and subjected to 16S rRNA sequencing. The diversity and species changes between developmental cohorts and age matched children with history of UTI was determined.

**Results:** A total of 75 subjects were recruited: 15 in each group. There was a clear evolution of the perineal microbiomes with development. There was a significant microbial disruption in girls with a history of UTI, irrespective of developmental milestone age group. The periurethral/perivaginal site displayed greater changes in microbiome structure than other sites in girls with a history of UTI.

**Discussion:** This pilot study evaluates the normal microbiome of the premenstrual girl at specific developmental milestones. Although the number of children per cohort was limited to 15, we observed statistical significance corresponding with developmental milestones. This study provides the first, culture independent delineation of the development of the perineal microbiome in girls. Furthermore, the sites closest to the site of infection appear to be more sensitive to antibiotic remodeling than those more distant. The factors that remodel the perineal microbiomes and predispose females, particularly girls, to UTIs (e.g., increase in uropathogen presence, absence of protective organisms) are unclear. Identification of specific signatures that increase susceptibility to UTI and their sequelae will improve patient care and promote personalized medicine.

## Introduction

Microbiota are recognized as an essential “second genome” that matures throughout childhood development and contributes to overall health (1–4). Dysbiosis of the well-characterized gastrointestinal microbiome is associated with multiple disease states (e.g., necrotizing enterocolitis, inflammatory bowel disease, irritable bowel syndrome, *Clostridium difficile*-mediated diarrhea, malnutrition, obesity, hypertension, liver disease) (3, 5), suggesting important roles of the microbiota in the modulation of disease susceptibility. To complicate matters more, environmental exposures, and host determinants can influence microbiome diversity, structure, and function in ways research has only begun to reveal. Specifically, while antimicrobial agents serve an important role treating serious bacterial infections, recent studies indicate that broad-spectrum antibiotics disrupt the mature microbiome. Antibiotic overuse is at epidemic proportions with one study reporting that 69% of children receive at least one antibiotic treatment before 2 years of age, with an average of 2.3 treatments per child by age 5 (6). The influence of early antibiotic exposure on microbiome maturation is understudied. Such liberal use of antibiotics at a critical time during development is particularly concerning for the potential impact on the natural development of a diverse microbiome as well as the development of antibiotic resistance within the microbiome, creating a “resistome” (7). The resistome has important therapeutic implications for treatment of future episodes of disease and the long-term health of children as they progress to adulthood.

Urinary tract infections (UTIs) are amongst the most common infections world-wide and hence, one of the most frequent reasons for antibiotic use, accounting for 1 in 5 of all antibiotics prescribed (8). A woman has a 50% chance of suffering at least one UTI in her lifetime, with a pooled prevalence of UTI in children of 8% (9). Of those diagnosed with an UTI, ~12–30% will suffer recurrent infections (10). The gastrointestinal microbiome provides a primary reservoir for uropathogens which can enter the urinary tract through direct contamination with fecal matter (11). In addition, healthy perineal (periurethral, perivaginal, and perianal) and vaginal microbiomes of adult women protect against UTIs, while dysbiosis of these sites increases susceptibility (11). Thus, there has been recent interest in the impact of the perineal microbiomes in the health of the genitourinary systems and how specific signatures might influence UTI risk in adult women. For example, the absence of *Lactobacillus* in the adult vagina is a risk factor for UTI while the probiotic *Lactobacillus* sp decreases susceptibility to recurrent UTI (11, 12).

There is strong evidence for the development of the core gastrointestinal microbiome as we mature (13). However, little is known regarding the maturation and segregation of the periurethral, perivaginal, and perianal microbiomes of girls. In addition, factors that modulate microbiome structure as well as signatures suggestive of health, disease, or susceptibility to UTI at these sites are unknown. Moreover, knowledge gaps remain for the role of habitat changes in the diversification of these spatially similar but distinct microbiome ecosystems. Microbiota at one site likely influences the microbiota at other sites due to the close proximity and the high potential for cross contamination. The environment and habitat of the perineum changes dramatically from exposure to fecal matter in diapers, transition to solid bowel movements, toilet training, hormonal changes in the epithelium associated with puberty, personal hygiene habits, contraception modalities, the physiochemical changes that occur during menstruation as well as the hormonal and tissue changes that occur following menopause. Each of these environmental and habitat changes likely translates into differences in the composition and stability of the flora; however, this is an understudied area of investigation.

Our overarching hypothesis is that the perineal microbiomes mature during childhood development and as observed with adult women, there will be specific signatures associated with UTI-prone and UTI-naïve girls. Our first aim of this study compared the evolution of the perineal flora coincident with accepted developmental milestones in healthy, UTI-naïve premenstrual girls to begin to delineate the normal female perineal microbiome. The second aim of this study compared the potential dysbiosis associated with local infection and antibiotic exposure on the perineal microbiomes of UTI-prone girls with age-matched healthy cohorts.

## Methods

The study protocol was approved by the Institutional Review Board for human studies (OHRP Assurance No. FWA00002860) at Nationwide Children's Hospital (NCH).

### Healthy Cohort of Females at Developmental Milestones

Healthy female subjects were recruited and consented/assented (as appropriate) at time of well-child visit to the NCH outpatient pediatric clinic. The subjects were divided into cohorts: “Newborn” (newborn-3 months of age), “Infant” (4–10 months of age), “Toddler” (2–6 years of age), and “Premenstrual” (7–12 years of age). The cohorts were selected based upon important milestones with potential habitat modifiers to the perineal microbiome: birth, introduction of solid foods to diet, toilet training, and moving from diapers to underwear, and girls entering into puberty but prior to onset of menarche, respectively. Subjects were included if they were presenting for well-child care without additional developmental or medical concerns. Subjects were excluded if they presented with an acute illness, any developmental concerns from either the family or the examining physician, known history of UTI or urinary/gastrointestinal tract abnormality other than constipation, known immunosuppression, current or recent (>3 months) antibiotic exposure, or had achieved menarche (menstruation).

### UTI Cohort of Females

Subjects in the UTI cohort, hereby also referred to as the UTI-prone cohort, were recruited and consented/assented (as appropriate) at time of outpatient visit to the NCH pediatric urology clinic as a referral for “UTI.” Subjects were included if the electronic medical record (EMR) confirmed at least one documented positive urine culture of at least >50,000 colony forming units per milliliter growth of a single bacterial organism (14). Chart review evaluated demographics, medical history with any imaging, number of documented positive urine cultures, and time from last antibiotic exposure/type of antibiotic prescribed. Subjects were excluded if presenting with a current concern for acute illness/UTI, current antibiotic use including antibiotic prophylaxis, known hydronephrosis, history of urinary tract surgery, known gastrointestinal tract abnormality other than constipation, known immunosuppression, or had achieved menarche. Subjects with known primary vesicoureteral reflux could be included, but only those without prior surgical intervention and/or those in whom surgical intervention was not indicated. Due to frequency of antibiotic exposure in this cohort, time from last antibiotic exposure was limited to >1 month. In order to capture demographic data, subjects were directly queried and the EMR reviewed to determine/validate date of last antibiotic exposure.

### Samples Selection and Collection

Our sampling of the perineal microbiome consisted of two swabs: (1) the periurethral/perivaginal area and (2) the perianal area. Due to anatomical constraints, particularly in the youngest subjects, sampling periurethral and perivaginal sites independently was not feasible, so a single swab of the perineurethral/perivaginal tissue sampled the flora of these sites (from now on referenced as the PUPV). We obtained a sample of the perianal flora as a site closely related to the PUPV with regard to anatomical distance and similar habitat changes during childhood. In addition, to enable comparison, we swabbed two other distinctly separate areas of the body upon which literature exists characterizing their microbiome: the oral cavity and retroauricular skin. Prior studies indicate that the oral microbiome composition matures with changes in dentition status. The age groups selected for our study also coincide with changes in dentition, enabling use of the oral cavity to represent a site with known changes in composition across cohorts and confirm appropriate choice of developmental milestones. Given that prior studies suggest that the retroauricular crease achieves maturity early in life, we selected this site to represent minimal shifts in composition across cohorts and thus compare what might be more systemic influences from that which is more localized. Dry mouth sponge swabs were used to collect samples from these 4 sites (“periurethral/perivaginal,” “perianal,” saliva,” and “retroauricular”). The first swab was a single swipe of the anterior oral cavity (“saliva”). The second swab was a single swipe across the retroauricular crease of the ear (“retroauricular”). The third swab sampled the combined periurethral/perivaginal area (heretofore referred to as PUPV), identified as the area of the labia minora from the clitoris down to the vaginal introitus. The fourth swab sampled the perianal area defined as the immediate epithelium around the anus. To reduce sampling variability, all patients were swabbed by one of two trained study staff. Swabs were immediately placed in a tube containing RNAlater (ThermoFischer, Waltham MA) and placed on dry ice. Samples were stored at −20°C until analysis.

### Next-Generation DNA Sequencing Swab Analysis

Frozen swabs were removed from RNA*later*, added to 180 μl of phosphate buffered saline and agitated for 45 min; following which the supernatant was removed and used for analysis. Bacterial DNA was isolated using a Qiagen DNA MiniAmp kit (Qiagen, Valencia, CA, USA) according to instructions. Two regions of the 16S rRNA genes were sequenced: V1–V3 (27F-515R) and V7–V9 (114F-317). The primers used for sequencing have been previously described (15). The 16s amplicons were quantified using the Quant-iT PicoGreen dsDNA reagent and kit (Invitrogen). Equimolar concentrations of each amplicon were pooled and sequenced on the MiSeq 2500 system (Illumina). Negative and positive controls (defined culture mixture) were used in all runs. The defined culture mixture is a stock mixture containing pre-quantified amounts of 11 oral bacteria. The species are *Streptococcus sanguis, S. mitis, S.oralis, Neisseria mucosa, Veillonella parvula, Actinomyces naeslundii, Prevotella intermedia, Porphyromonas gingivalis, Tannerella forsythia, Fusobacterium nucleatum* and *Filifactor alocis*. Two primer pairs were used since each primer pair is capable of detecting a range of genera that the other fails to recover. Together they allow the recovery of a wider range of the microbiome than is possible with a single primer pair alone. However, some genera are detected by both primer pairs. Thus, to prevent overcounting, the number of sequences assigned to an operational taxonomic unit (OTU) by both primer pairs was reduced by half. Averaging of the PCR products were carried out as previously described (15) using the implementation in the PhyloToAST software suite (16). Analyses were conducted using QIIME1.9.0 (17) and PhyloToAST. The sequences were binned by sample and aggregated. *De novo* OTUs were identified. Sequences were clustered into distinct OTUs at 97% similarity using the UCLUST method (18). Chimeric sequences were depleted using ChimeraSlayer (v. 1.9.0, identify_chimeric_seqs.py). Sequences with an average quality score of 30 over a sliding window of 50 bp and length >200 bp were assigned a taxonomic identity by alignment to the Greengenes database (http://greengenes.lbl.gov) using the Blastn algorithm at 97% identity. Alpha (within-group) and beta (between-group) diversity were computed. Since emergent evidence does not support rarefying the microbiome to compensate for sequencing effort (19), we used cumulative sum scaling (CSS) normalization from the Bioconductor package metagenomeSeq. Both phylogenetic (UniFrac) and non-phylogenetic (Bray-Curtis) distance matrices were utilized to estimate beta diversity. Analyses was performed on distance matrices, and significance of clustering was interrogated using Adonis with 999 permutations. Plots were generated by the R package ggplot. A core microbiome was identified when species were present in at least 80% of patients in each group using get_core_ids.py script (https://github.com/akshayparopkari/kadambari/blob/master/python/get_core_ids.py). Species abundances were not considered for inclusion into the core microbiome. Sequences can be accessed from the Sequence Read Archive of the NCBI using the submission number SRA PRJNA670727.

### Statistical Analysis

Beta diversity was measured with Adonis and ANOSIM tests to estimate statistical differences between groups. The Bioconductor package for R, *DESeq2*, was used to perform differential composition of the microbiome structure (20). This function uses a negative binomial distribution of raw counts to estimate between-group differences, while accounting for sampling effort (library size) and dispersion of each category (taxon or functional gene). p-values were adjusted for multiple testing (FDR < 0.1, FDR-adjusted Wald Test).

D'Agostino-Pearson omnibus normality test was performed to determine if samples were parametric. Depending on number of cohorts being compared, we analyzed differences in demographics using Student's *t* test or Analysis of Variance when the samples were parametric and Mann-Whitney *U*-test or Kruskal-Wallis *H*-test when non-parametric. In cases of unequal sample sizes, a Welch's *t*-test was used. A *p*-value of < 0.05 was considered significant.

## Results

### Demographics of Healthy Girls

Fifteen subjects for each of the 4 cohorts were included in our healthy cohort of females, resulting in 60 total healthy subjects recruited with no history of UTI and no recent antibiotic exposure (>3 months). Consistent with our Columbus, Ohio community, we recruited a diverse group of subjects with 63% non-Hispanic Black, 21% non-Hispanic White, and 15% other. As expected, the cohorts differed significantly in age and dentate status, but otherwise were not significantly different in other reported demographics (Table 1).

**Table 1:** Healthy cohort demographics.

	**Newborn controls (*n* = 15)**	**Infant controls (*n* = 15)**	**Toddler controls (*n* = 15)**	**Premenstrual controls (*n* = 15)**	**Overall controls (*n* = 60)**	***P***
Median age years (mean; range)	0.05 (0.08; 0.02–2.7)	0.55 (0.56; 0.36–0.85)	4.08 (4.37; 2.68–6.89)	9.98 (9.89; 7.09–11.90)	1.8 (3.7; 0.02–11.9)	<0.0001
Smoke exposure (%)	6 (40)	7 (47)	3 (20)	6 (40)	22 (37)	0.4680
**INFANT DIET**						
History of being Breast fed (%) Breast + Formula (% of Breast fed) Formula only fed (%) Unknown how fed (%)	6 (40) 2 (33) 9 (60) 0 (0)	4 (26) 3 (75) 11 (73) 0(0)	8 (53) 4 (50) 5 (33) 2 (13)	3 (20) 2 (66) 9 (60) 3 (20)	21(35) 11 5 (8)	0.1980
Pre-dentate (%)	15 (100)	10 (67)	0 (0)	0 (0)	25 (42)	<0.0001
**RACE (%)**						
Caucasian African American Hispanic Asian Arabic Multiracial Unknown	3 (20) 8 (53) 1 (7) 0 (0) 1 (7) 2 (13) 0 (0)	3 (20) 9 (60) 1 (7) 0 (0) 0 (0) 2 (13) 0 (0)	3 (20) 10 (67) 0 (0) 1 (7) 0 (0) 1 (7) 0 (0)	2 (13) 11 (73) 1 (7) 0 (0) 0 (0) 0 (0) 1 (7)	11 (18) 38 (63) 3 (5) 1 (2) 1 (2) 5 (8) 1 (2)	0.8908
**METHOD OF BIRTH**						
Vaginal birth (%) C-section (%) Unknown (%)	13 (87) 2 (13) 0 (0)	13 (87) 2 (13) 0 (0)	11 (73) 2 (13) 2 (13)	10 (67) 2 (13) 3 (20)	47 (78) 8 (13) 5 (8)	0.9936

### The Microbiome Signatures of the Saliva, Retroauricular, Perianal, and Periurethral/Perivaginal Sites Significantly Differ From Each Other

**Figure 1:**
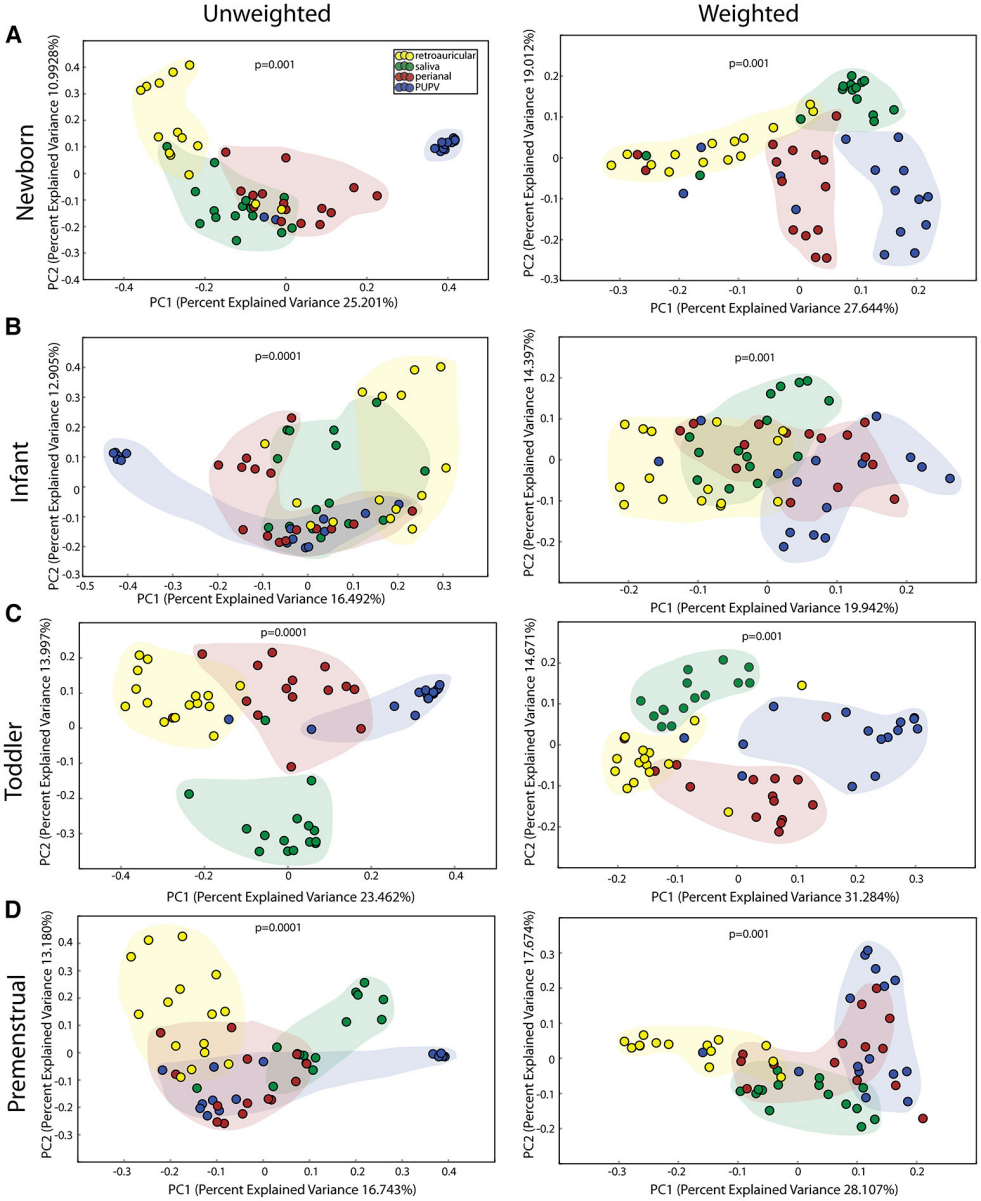
Comparison of microbiomes by site in the healthy cohorts. Unweighted UniFrac profile evaluating for differences in community members and weighted UniFrac profile of community structure for **(A)** newborn cohort, **(B)** infant cohort, **(C)** toddler cohort, and **(D)** premenstrual cohort. Each data point represents the bacterial community of a given subject for the retroauricular (yellow), saliva (green), perianal (red), and periuretheral/perivaginal (PUPV, purple) sites. Color clouds are intended for ease of visualization and do not provide any statistical value. Statistical significance was determined by ANISOM analyses.

We observed statistically significant different populations for community membership (unweighted), and community structure (weighted) for all 4 sites within each cohort (Figure 1). Consistent with other studies (21), these data indicate that microbiota is site specific for each of the developmental groups.

### The Acquisition and Maturation of Microbiota Is Significant in the Saliva

**Figure 2:**
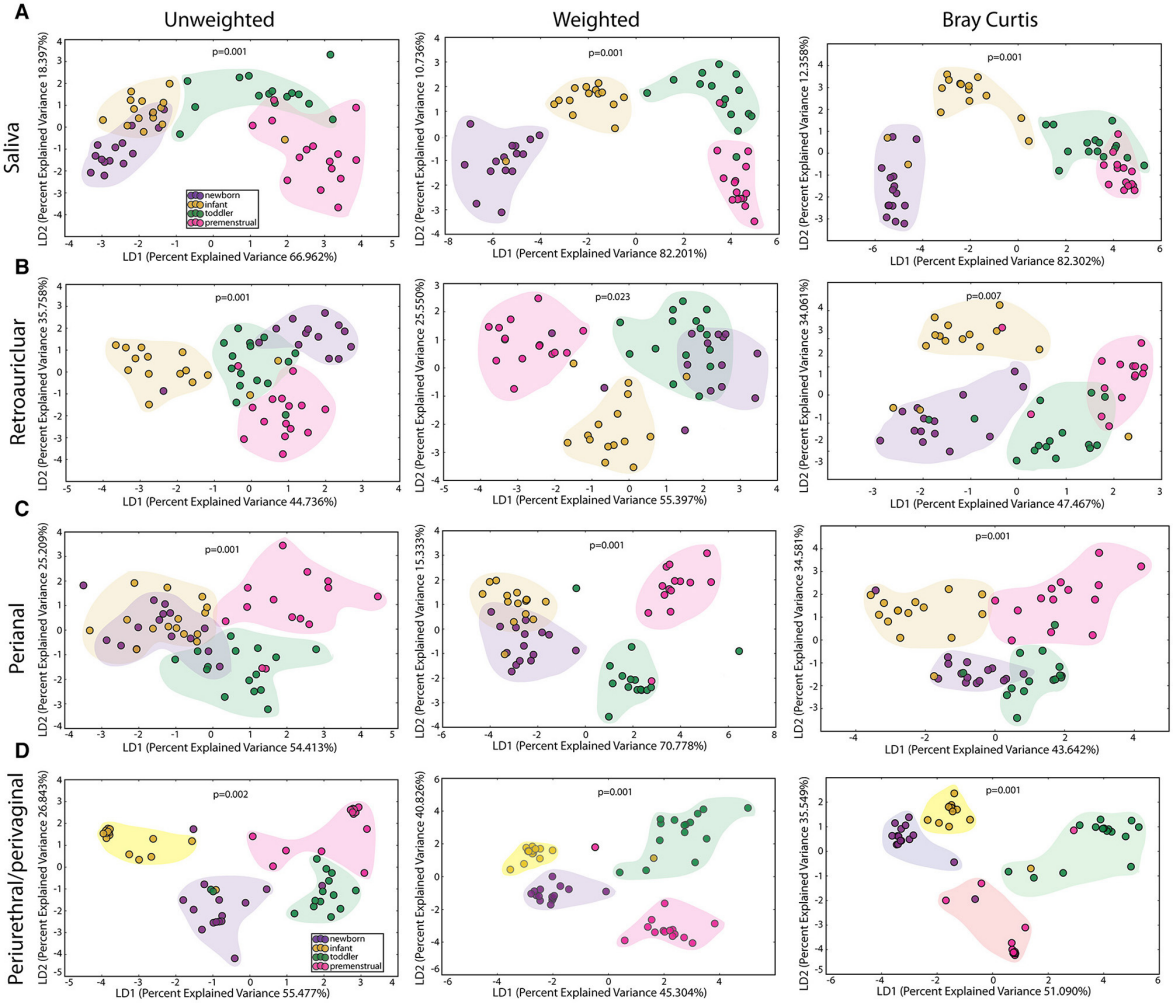
Comparison of the microbiomes by age in the healthy cohorts. Unweighted UniFrac profile evaluating for differences in community members, weighted UniFrac profile of community structure, and the Bray-Curtis dissimilarity index determined for the **(A)** saliva, **(B)** retroauricular, **(C)** perianal, and **(D)** periurethral/perivaginal sites. Each data point represents the bacterial community of a given subject in the newborn (purple), infant (yellow), toddler (green), and premenstrual (fuchsia) cohort. Color clouds are intended for ease of visualization and do not provide any statistical value. Statistical significance was determined by ANISOM analyses.

We observed a statistically significant difference for community membership (unweighted), community structure (weighted) as well as phylotype-based compositional dissimilarity (Bray-Curtis) amongst developmental age groups in the saliva (Figure 2A), indicating that these groups differed both in presence or absence of lineages, as well as in the relative abundances of lineages within communities. The clustering of the toddlers (green clouds) and premenstrual group (fuchsia clouds) from the newborn (purple clouds) and infant (yellow clouds) is consistent with the presence of dentition in both of the older age groups.

**Figure 3:**
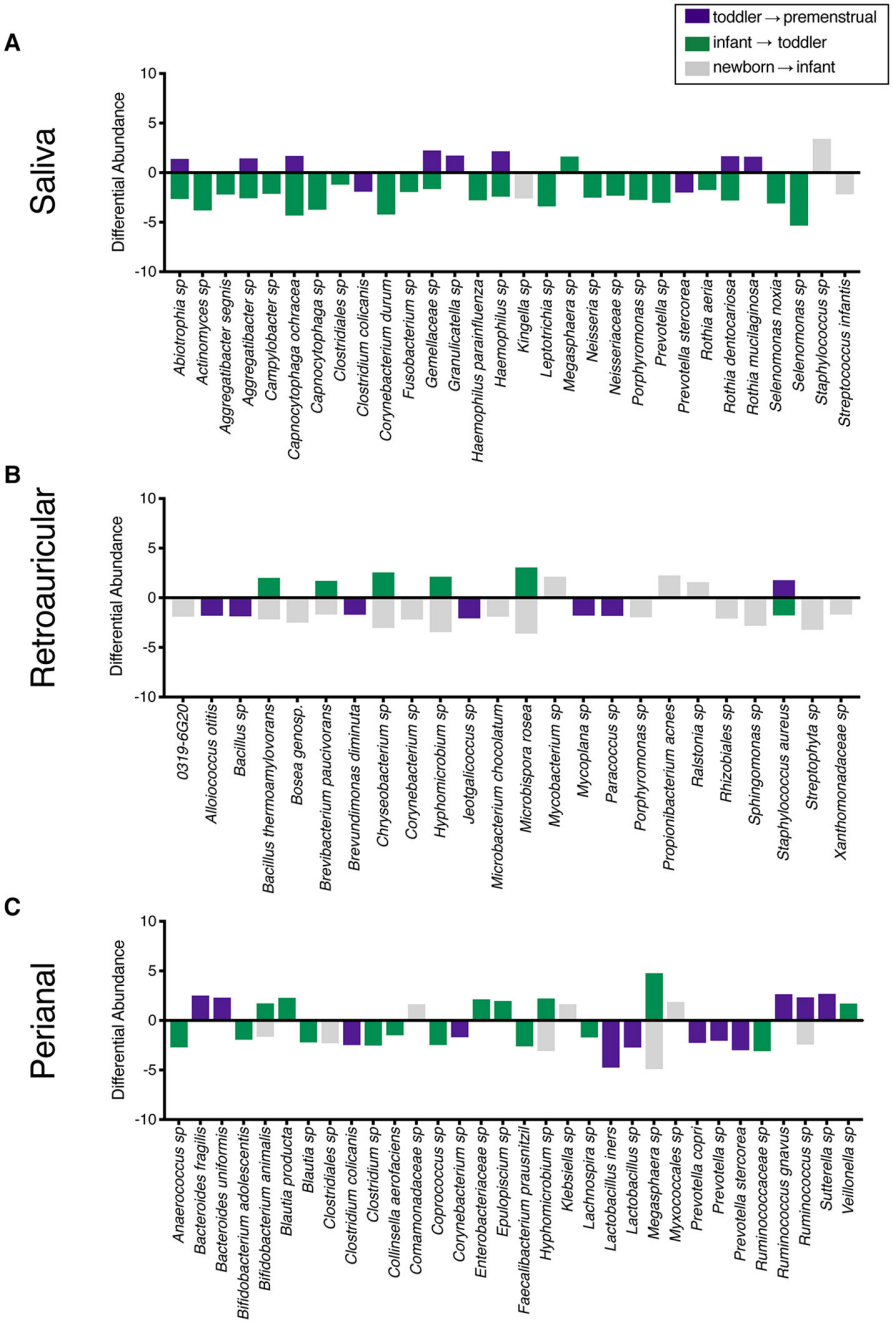
Changes in species observed between healthy cohorts. List of OTUs with a statistically significant log^2^ fold change >2 are indicated for the comparison of newborn to infant (gray), infant to toddler (green), and toddler to premenstrual (purple) for the **(A)** saliva, **(B)**, retroauricular, and **(C)** perianal sites.

To determine the maturation of the microbiome at the genus/species level, we identified OTUs that exhibited a statistically significant log^2^ fold change in relative abundance of >1.5 fold across the age cohorts. We evaluated the changes from newborn to infant (gray), infant to toddler (green), and toddler to premenstrual (purple) (Figure 3A). The majority of the changes in the differential abundances of OTUs for the saliva occurred in the comparison of the infant and toddler cohorts, consistent with the major habitat change of the oral cavity being the eruption of detention between infancy and toddler ages (22). In virtually all cases, there was a loss of OTUs in the toddler cohort. Of the species that were lost, *Aggregatibacter* sp and *Porphyromonas* sp are associated with periodontal disease (Figure 3A). The fewer changes observed when comparing the newborn to infant and the toddler to premenstrual cohorts reflect the similarity in dentition status between these cohorts.

### The Acquisition and Maturation of Microbiota Is Significant at the Retroauricular Site

The retroauricular skin had previously been considered a stable site that matures early in life (23, 24). However, we observed a statistically significant difference between developmental groups in the community membership (unweighted), community structure (weighted), as well as phylotype-based compositional dissimilarity (Bray-Curtis) at the retroauricular site (Figure 2B). The overall changes in species was modest; we observed a loss of 14 OTUs between the newborn and infant cohorts (Figure 3B). There were increases in 5 OTUs in the transition from infant to toddler with the loss of 6 OTUs in the transition from toddler to premenstrual cohorts, suggesting that the core skin microbiome is acquired early but continues to exhibit minor changes as girls age.

### The Acquisition and Maturation of Microbiota Is Significant at the Perianal Site

The perianal site is contiguous with the colon and likely experiences continuous seeding from the shed gastrointestinal microbiome. As with the other sites evaluated here, we observed a statistically significant difference in the community membership (unweighted), community structure (weighted), as well as phylotype-based compositional dissimilarity (Bray-Curtis) at the perianal site for all developmental groups (Figure 2C). In contrast to the saliva and retroauricular microbiomes where a single milestone appears to contribute to maturation, the perianal site appears to exhibit continual changes with the acquisition and loss of similar numbers of OTUs at each milestone (Figure 3C). Interestingly, despite being present in the earlier cohorts, there was a loss of some known beneficial species (e.g., *Bifidobacterium, Lactobacillus*) from the perianal site in the premenstrual cohort.

### The PUPV Microbiome Experiences Shifts in Bacterial Communities Between Developmental Cohorts

The PUPV sites are exposed to similar habitat changes as the perianal site, but also experience additional changes during the transition to puberty. As with all the other sites studied here, we observed significant differences in the overall community membership (unweighted), community structure (weighted), as well as phylotype-based compositional dissimilarity (Bray-Curtis) (Figure 2D) across age cohorts.

**Figure 4:**
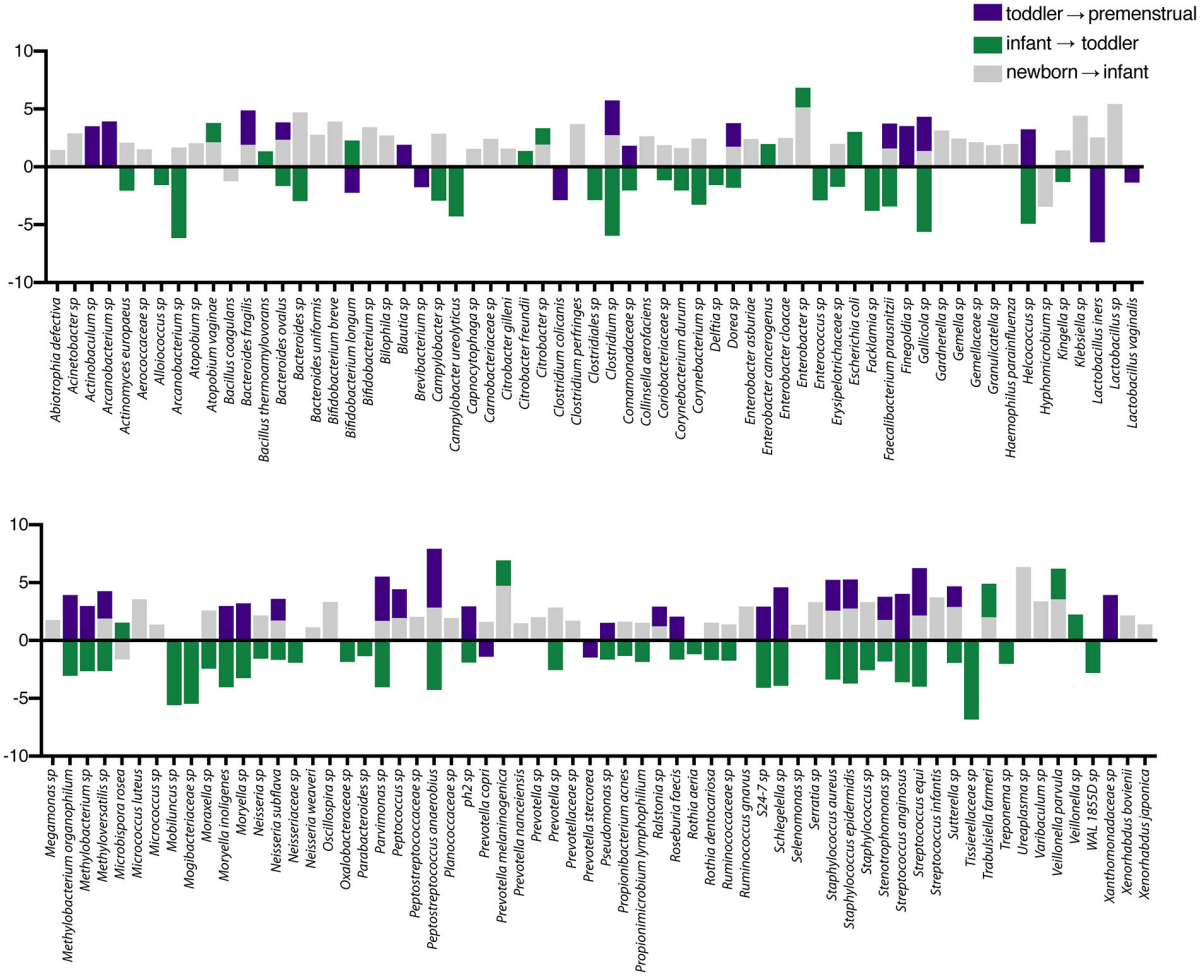
Changes in species observed between healthy cohorts at the periurethral/perivaginal site. List of OTUs with a statistically log^2^ fold change >2 are indicated for the comparison of newborn to infant (gray), infant to toddler (green), and toddler to premenstrual (purple).

**Figure 5:**
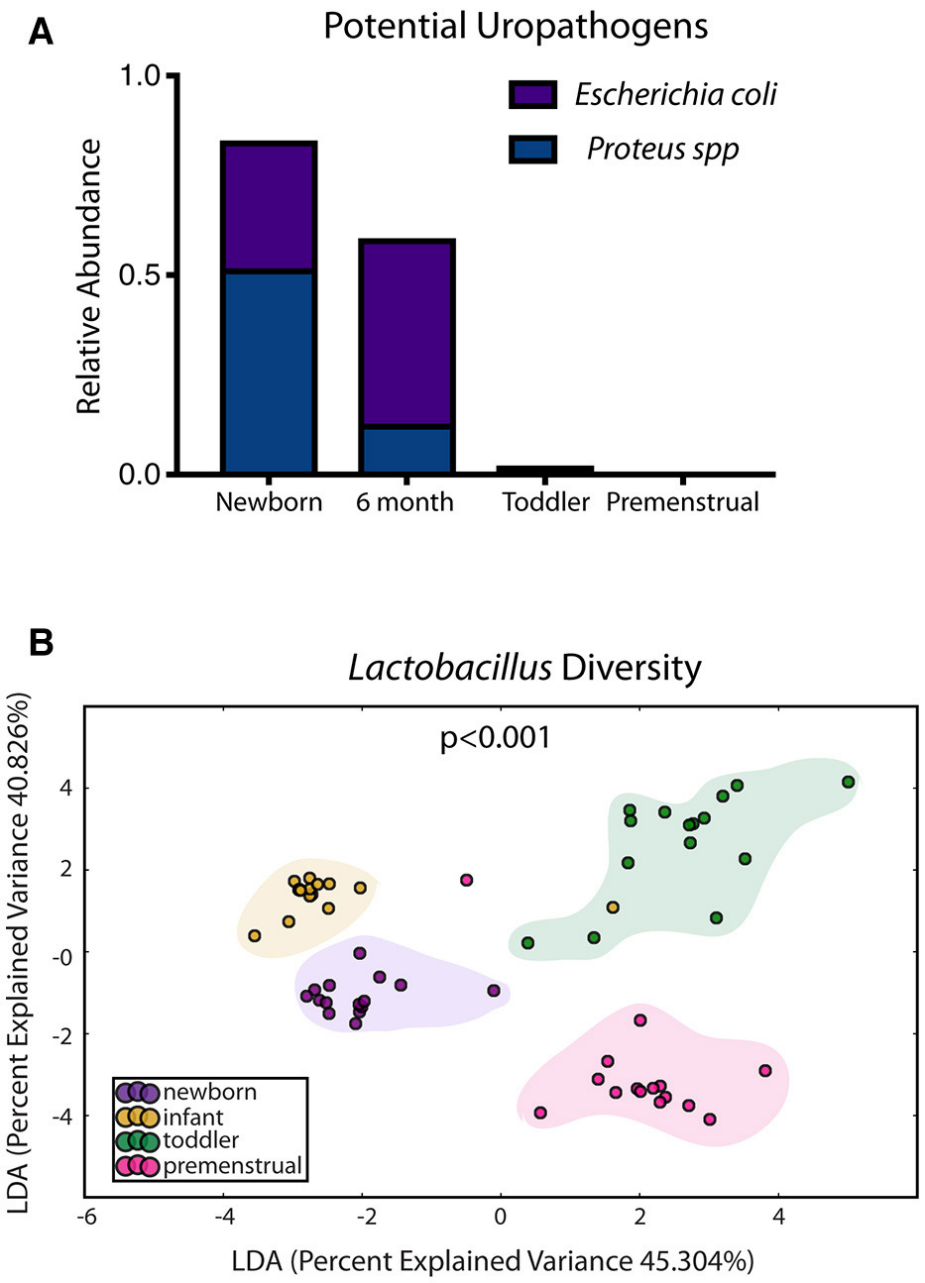
Known organisms that promote UTI-susceptibility exhibit shifts in population at the periurethral/perivaginal site as girls age. **(A)** The relative abundance of two potential uropathogens decrease within the microbiome as girls age. **(B)** Statistically significant shifts in the community membership for *Lactobacillus* sp are observed in each healthy aged newborn (purple), infant (yellow), toddler (green), and premenstrual (fuchsia) cohort.

In contrast to the other sites, the PUPV site appears more plastic throughout childhood as we did not observe a dominant milestone for the changes at the genus/species level. We observed changes in 127 OTUs within the PUPV microbiome throughout childhood (Figure 4). We observed acquisition of OTUs in the infant and premenstrual cohorts, with primarily a loss of OTUs in the toddler cohort. Some of the organisms were significantly changed in the transition between milestones (e.g., *Acinetobacter* sp, *Campylobacter ureolyticus, Blautia* sp) while others appear to continually fluctuate during all milestones (e.g., *Gallicola* sp, *Peptostreptococcus anaerobiu*s, *Staphylococcus epidermidis*). We observed several noteworthy changes. Toddlers had considerably more *E. coli* in their PUPV as compared to the other cohorts, particularly the infants. We observe a sequential decrease in the overall presence of potential uropathogens from the PUPV sites, registering highest in the newborn and lowest in the premenstrual cohorts (Figure 5A). The infant group demonstrated increases in various *Clostridium* sp*, Gardnerella, Staphylococcal* sp, *Bacterioides fragilis*, and *Bifidobacterium* sp (Figure 4). We observe fluctuations in the relative levels of *L. iners* in the cohorts with a greater than log^2^ fold reduction in the PUPV of the premenstrual group (Figure 4). In addition, we observe a significant difference in the community membership of the *Lactobacillus* species across the cohorts by age (Figure 5B).

### The Perianal and PUPV Sites Diverge Early

**Figure 6:**
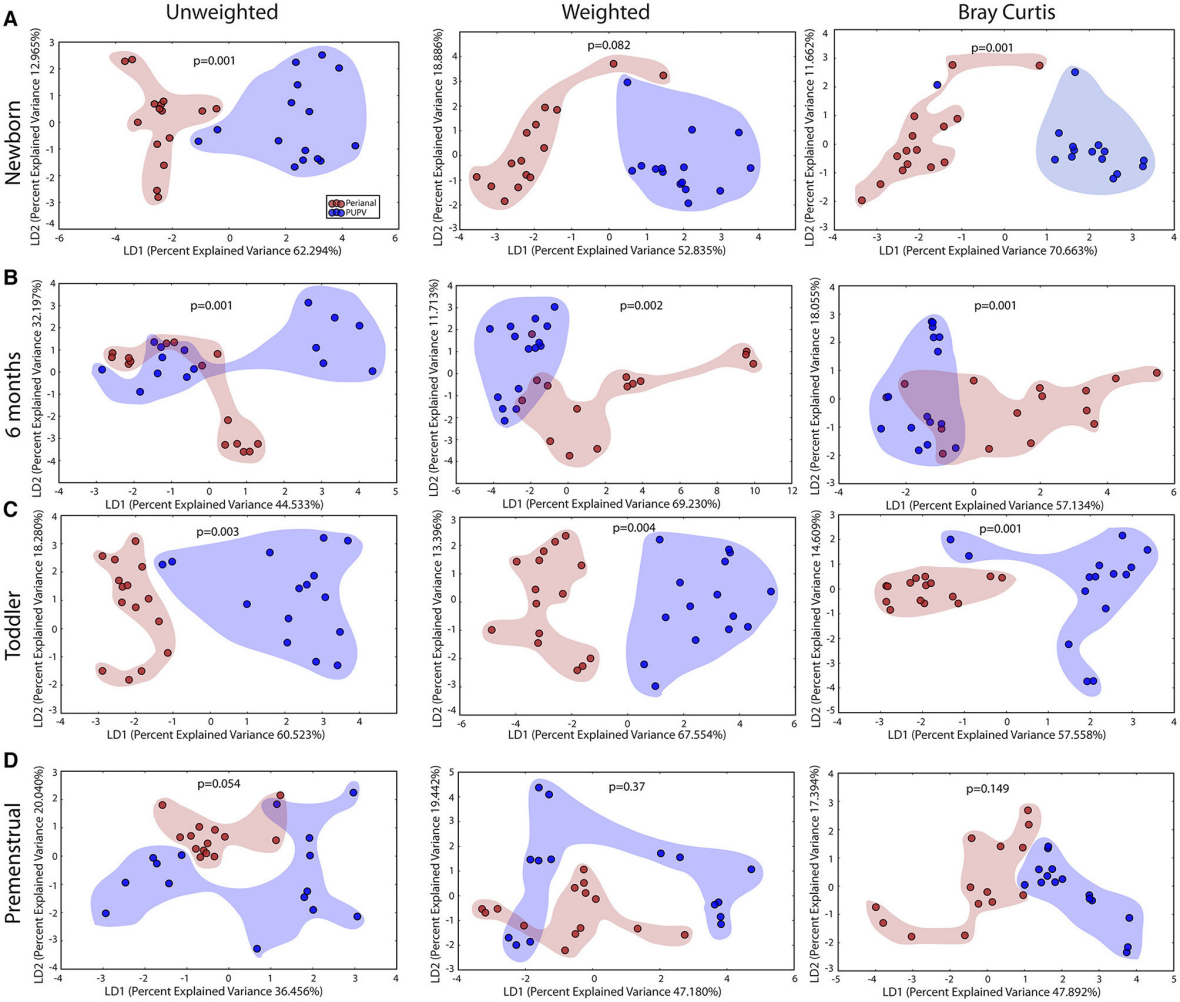
The perianal and periurethral/perivaginal microbiomes diverge from each other as early as the newborn. Unweighted UniFrac profile evaluating for differences in community members and weighted UniFrac profile of community structure for **(A)** newborn cohort, **(B)** infant cohort, **(C)** toddler cohort, and **(D)** premenstrual cohort. Each data point represents the bacterial community of a given subject for the perianal (red) and periuretheral/perivaginal (purple) sites. Color clouds are intended for ease of visualization and do not provide any statistical value. Statistical significance was determined by ANISOM analyses.

Despite the close anatomic proximity that could enable cross contamination between the perianal and PUPV sites, a statistically significant difference between these sites was observed when comparing all sites across all cohorts (Figure 2D). To determine the contribution of the shared habitat to the bacterial communities, the perianal and PUPV sites were compared directly (Figure 6). The community membership and phylotype-based compositional dissimilarity were also significantly different in the newborn, suggesting that these sites have individual pioneer species (Figure 6A). The distinction of these sites was maintained in the infant and toddler cohorts (Figures 6B,C). Interestingly, the perianal and PUPV microbiomes were not significantly different in the premenstrual cohort (Figure 6D).

### Demographics of the UTI-Prone and UTI-naïve Cohorts

To evaluate the potential contribution of UTI as well as antibiotic exposure on the structure of the microbiomes, we enrolled 15 female subjects with a history of UTI. We then age-matched this UTI-prone cohort with girls from our healthy cohorts (UTI-naïve). A comparison of the demographics between these two groups showed significant differences in race and birth history (Table 2). These differences prompted further investigation to determine the contribution of these external modifiers on their microbiomes. We did observe a significant difference between races for community membership and phylotype-based compositional dissimilarity at the perianal site (Supplementary Figure 1). However, no significant differences between races were observed at the oral, retroauricular, or PUPV sites. Unfortunately, due to the absence of cesarean section delivery in the UTI-naïve cohort, we were unable to determine a potential contribution of birth mode to the microbiome. Lastly, EMR review revealed that 5 subjects in the UTI-prone cohort (*n* = 15) had a documented antibiotic allergy; whereas, no antibiotic allergies were noted in the entire healthy cohort (*n* = 60).

**Table 2:** UTI-prone vs. UTI-naive demographics.

	**UTI (*n* = 15)**	**Age-matched controls (*n* = 15)**	***P***
Median age years (mean; range)	6.33 (5.89; 0.28–12.37)	5.51 (5.83; 0.20–11.90)	0.9682
Smoke exposure (%)	4 (27)	7 (47)	0.2557
**INFANT DIET**			
History of being Breast fed (%) Breast + Formula (% of Breast fed) Formula only fed (%) Unknown how fed (%)	5 (33) 2 (40) 5 (33) 5 (33)	4 (27) 3 (75) 11 (73) 0 (0)	0.2338
Pre-dentate (%)	2 (13)	2 (13)	1.000
**RACE (%)**			
Caucasian African American Multiracial Unknown	14 (93) 0 (0) 1 (7) 0 (0)	1 (7) 12 (86) 1 (7) 1 (7)	<0.0001
Method of birth			0.0169
Vaginal birth (%) C-section (%) Unknown (%)	9 (60) 6 (40) 0 (0)	14 (93) 0 (0) 1 (7)	
Median last antibiotic in months (mean; range)	1.6 (3.7; 1–16.5)		
Median number UTIs (mean; range)	2 (3.6; 1–14)		
Median time from last UTI in months (mean; range)	2.7 (6.9; 0.9–23.8)		
VCUG performed Vesicoureteral reflux present (% of those with VCUG) Dilating VUR present (% of those with VUR)	9 4 (44) 1 (25)		
Allergies to antibiotics	5		

### Microbiome Alterations in the Saliva, Retroauricular, and Perianal Sites Between the UTI-Prone and UTI-naïve Cohorts

**Figure 7:**
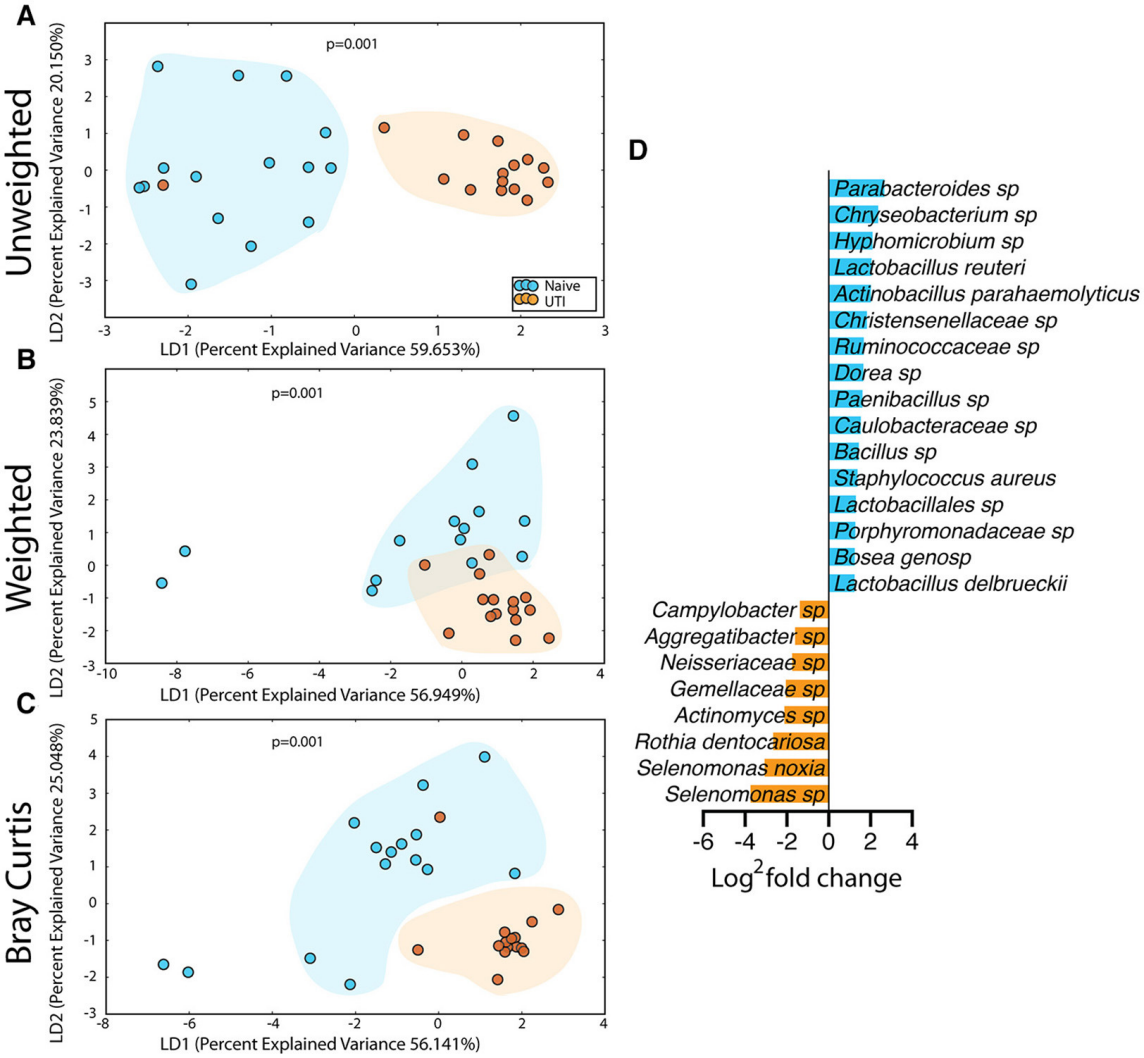
Statistically significant differences are observed in the salivary microbiome of UTI-prone girls (orange) compared to UTI-naïve girls (teal). **(A)** Unweighted UniFrac profile evaluating community membership. **(B)** Weighted UniFrac profile evaluating for differences community structure. **(C)** Bray-Curtis dissimilarity index. **(D)** Deseq list of statistically significant bacterial OTUs with a statistically significant log^2^ fold change >2. Color clouds are intended for ease of visualization and do not provide any statistical value. Statistical significance was determined by ANISOM analyses.

**Figure 8:**
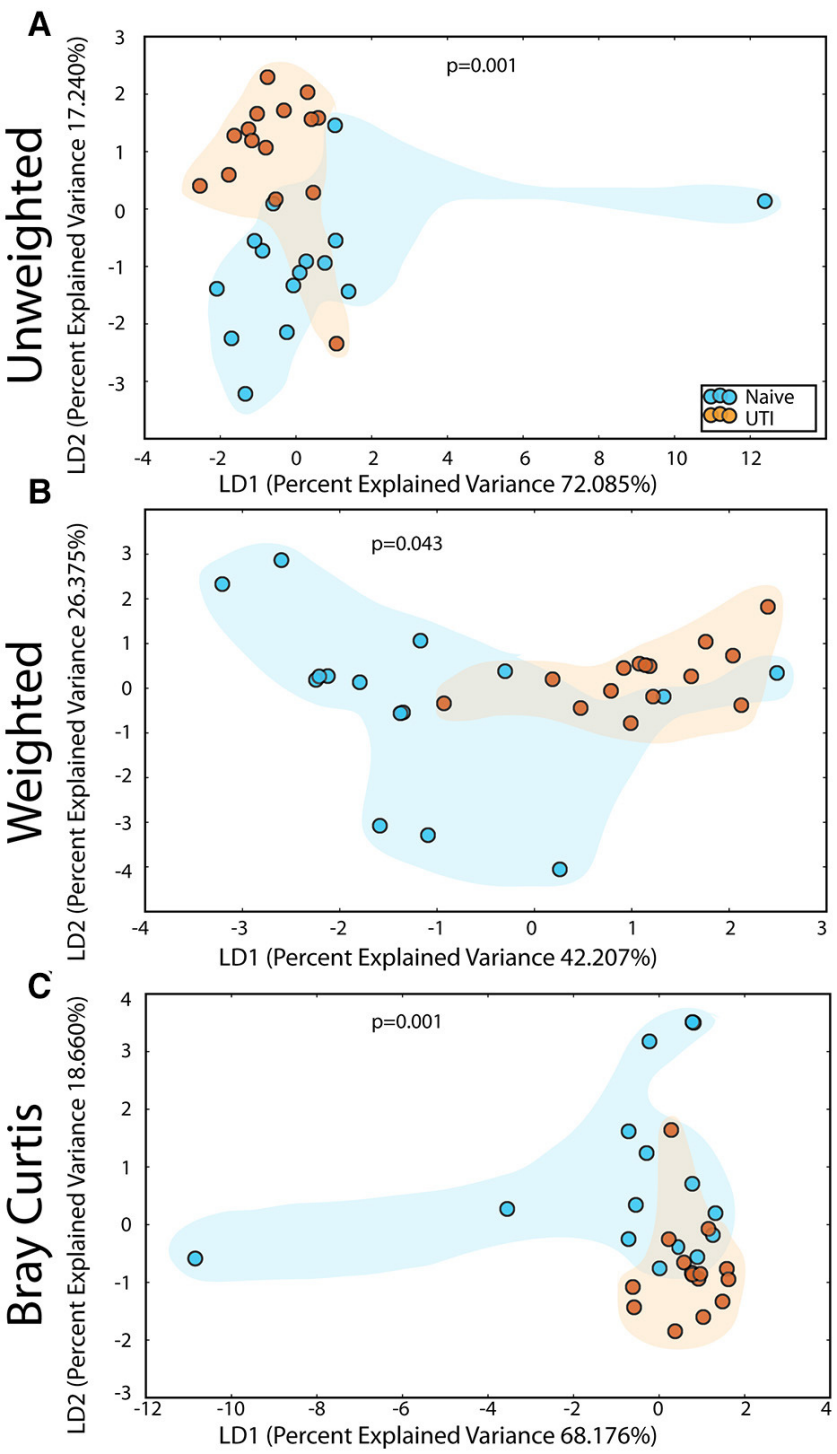
Statistically significant differences are observed in the retroauricular microbiome of UTI-prone girls (orange) compared to UTI-naïve girls (teal). **(A)** Unweighted UniFrac profile evaluating community membership. **(B)** Weighted UniFrac profile evaluating for differences community structure. **(C)** Bray-Curtis dissimilarity index. Color clouds are intended for ease of visualization and do not provide any statistical value. Statistical significance was determined by ANISOM analyses.

**Figure 9:**
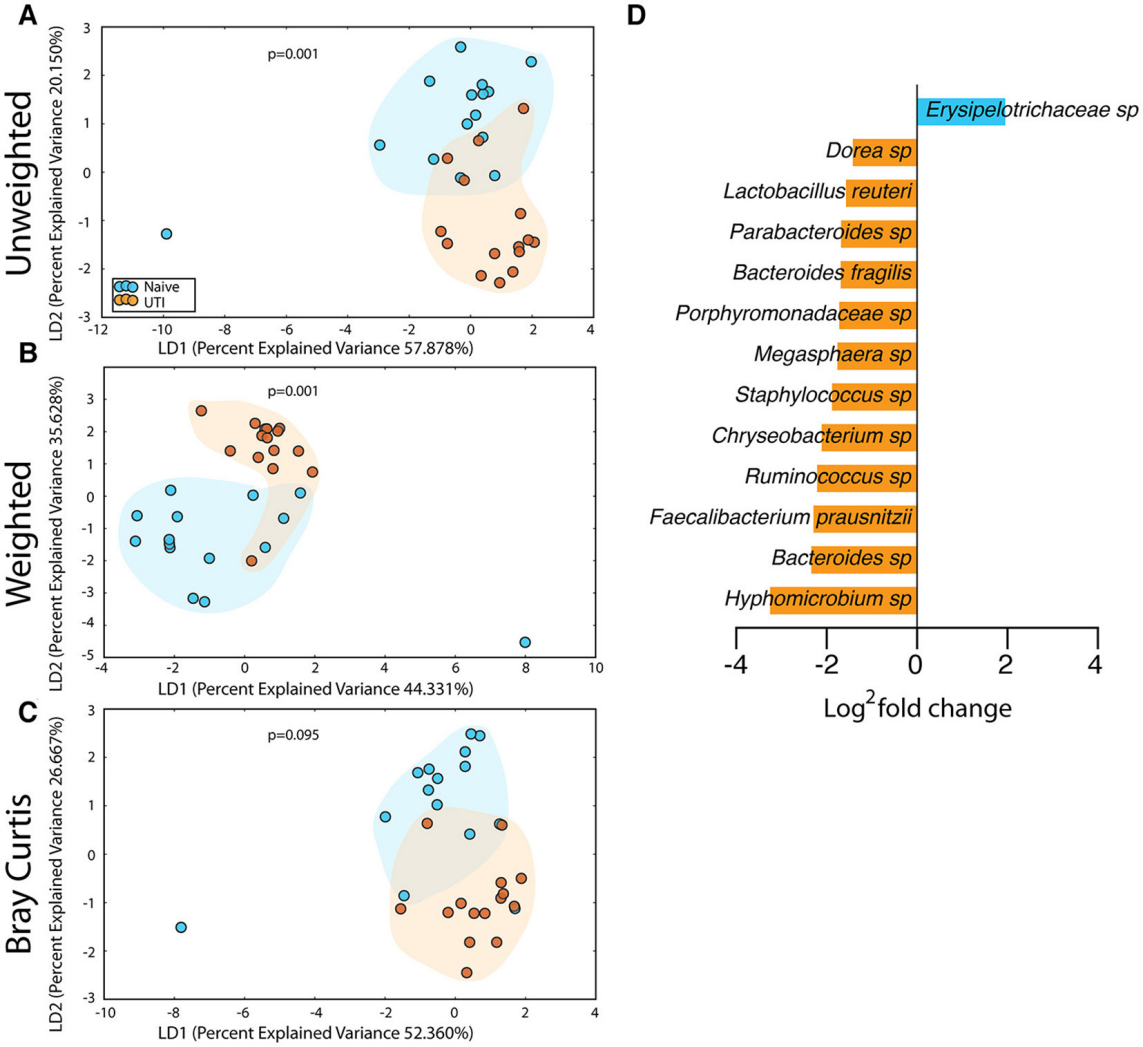
Statistically significant differences are observed in the perianal microbiome of UTI-prone girls (orange) compared to UTI-naive girls (teal). **(A)** Unweighted UniFrac profile evaluating community membership. **(B)** Weighted UniFrac profile evaluating for differences community structure. **(C)** Bray-Curtis dissimilarity index. **(D)** Deseq list of statistically significant bacterial OTUs with a statistically significant log^2^ fold change >2. Color clouds are intended for ease of visualization and do not provide any statistical value. Statistical significance was determined by ANISOM analyses.

The salivary microbiome of the UTI-prone cohort (orange) differed significantly from the salivary microbiome of the UTI-naive cohort (blue) (Figure 7). We observed an increase in 16 OTU and a loss of 8 OTU from the UTI-prone girls (Figure 7D). The retroauricular site also demonstrated a significant difference between the UTI-prone and UTI-naïve subjects (Figure 8). At the perianal site, we also observed a significance difference in the community membership (unweighted) and community structure (weighted), analyses (Figures 9A,B). Curiously, the phylotype-based compositional dissimilarity (Bray-Curtis) showed no significant difference (Figure 9C). In addition, the changes in OTU at this site were modest with a loss of one OTU and a gain in 12 OTU in the UTI-prone girls (Figure 9D). Given the known association of UTI infection and antibiotic exposure, fewer changes could suggest this site experiences less antibiotic related affects or that this site experiences less infection induced affects. In all, the history of UTI is associated with modification of the microbiome at these sites in young girls.

### Microbiome Alterations of the PUPV Site Between the UTI-Prone and UIT-naïve Cohorts

**Figure 10:**
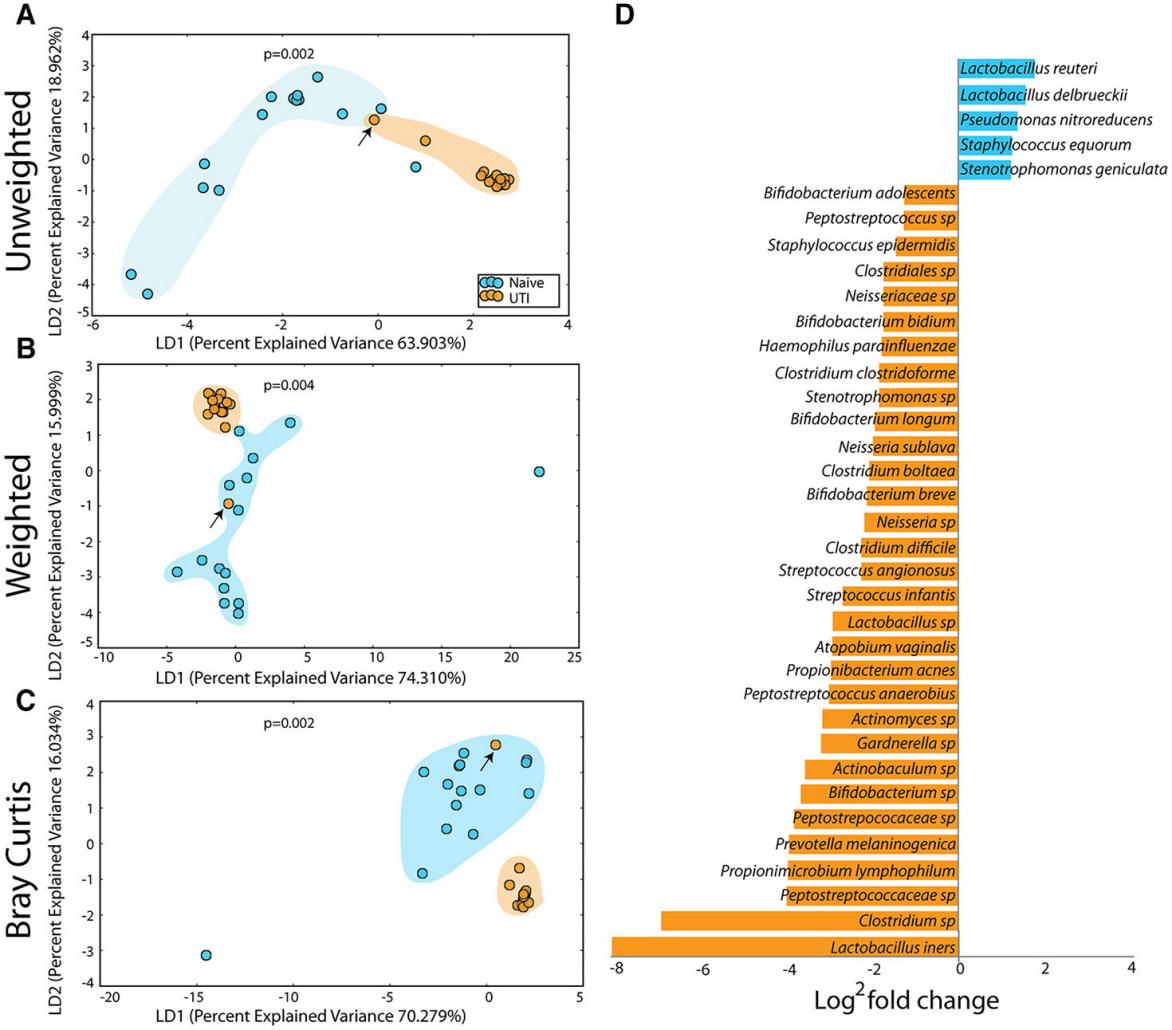
Statistically significant differences are observed in the periurethral/perivaginal microbiome of UTI-prone girls (orange) compared to UTI-naïve girls (teal). **(A)** Unweighted UniFrac profile evaluating community membership. **(B)** Weighted UniFrac profile evaluating for differences community structure. **(C)** Bray-Curtis dissimilarity index. **(A–C)** The black arrow demonstrates the UTI-prone patient who was farthest out from last antibiotic exposure (16.5 months) compared to other UTI-prone girls. **(D)** Abbreviated Deseq list of statistically significant bacterial OTUs with a statistically significant log^2^ fold change >2 (see Supplementary Figure 2 for full list). Color clouds are intended for ease of visualization and do not provide any statistical value. Statistical significance was determined by ANISOM analyses.

The PUPV microbiome of the UTI-prone cohort (orange) differed significantly from the PUPV microbiome of the UTI-naive cohort (blue) (Figure 10). Additional analyses also reveal a statistically significant difference in the phylotype-based compositional dissimilarity (Bray-Curtis) of the UTI-prone cohort compared to UTI-naive. One UTI subject clustered away from the UTI cohort and more similarly to the UTI-naïve cohort (see black arrow). Upon further review, she exhibited the longest time from her last antibiotic exposure (16 months) and her second UTI (19 months).

A representative subset of the changes in OTUs is presented (Figure 10D). Overall, we observed a significant increase in 15 OTUs and a significant loss in 81 OTUs from the UTI-prone girls (Supplementary Figure 2). The UTI-naïve girls demonstrated more abundance in important beneficial taxa like *Bifidobacterium* sp and *Lactobacillus* sp than their UTI-prone counterparts. We also observed a log^2^ 8-fold loss of *L. iners*. The only exception was *L. reuteri*, a species often found in probiotics, which was more abundant in the UTI-prone cohort. Compared to the saliva and perianal sites (Figures 7, 9), the PUPV site (Figure 10) experienced more changes in OTUs, suggesting the disease exerts a specific local effect on microbiome composition or this site is more susceptible to antibiotics.

## Discussion

The perineum of developing children, particularly girls, experiences significant physiochemical changes that would seemingly influence microbiome maturation. The environment changes dramatically from exposure to waste matter in diapers, changes in fecal consistency with the transition to solid food, changes in hygiene habits with toilet training and initiation of menstruation, and hormonal changes associated with puberty. While one would hypothesize that each of these stages translates into significant differences in the composition and stability of the flora, our knowledge of the normal acquisition, and composition of the perineal microbiome in the premenstrual female has been limited until now. Our findings found significant changes in the diversity of the microbiome in all sites tested at each developmental milestone evaluated. The penultimate goal of this work is to identify specific microbiome signatures that confer either significant risk or benefit in terms of UTI susceptibility and identify crucial periods of time when that microbiome may be the most vulnerable to modulation. That is an unfeasible goal for a small pilot study; however, our study does provide preliminary data describing an evolution of the perineal microbiomes and a disruption most acutely observed at the PUPV site in the setting of previous UTI.

Our study is the first to approach this problem in pediatrics utilizing culture-independent approaches like next-generation sequencing; the available studies previously published rely on culture-based techniques. Those results are limited, only reporting the presence of potential pathogens in health and during vulvovaginitis in prepubescent girls (25, 26); and a more recent study which went further to associate the presence of potential periurethral pathogens and vulvovaginitis with an increase in UTIs in girls (27). From studies in adult women, the normal flora at the PUPV sites is known to modulate UTI susceptibility by excluding potential uropathogens (23). Our study builds on that earlier work by describing the composition of the pediatric perineal microbiome down to the species level and evaluating potential links from individual species to UTI susceptibility. In terms of potential uropathogens, our results demonstrated a decrease in potential uropathogens as a girl ages. This tracks with UTI rates that drop after infancy and do not increase again until sexual debut (28). At a species level, we found an abundance of *Lactobacillus sp* in our toddler patients and an actual reduction in expression of *L. iners* and *L. vaginalis* in our premenstrual cohort. Such fluctuations suggest an interesting evolution of *Lactobacillus* sp residing in the PUPV region and deserves more study to better understand its role in promoting genitourinary health. Additionally, recent studies suggest that *L. iners* predominates when the vaginal flora is transitioning from dysbiotic to normal (29). Our UTI-prone patients demonstrated an alarming log^2^ reduction in *L*. iners and *Lactobacillus sp*, clearly suggesting some dysbiosis in this patient population.

Continuing our review of species levels changes between our cohorts, we found some associations of bacterial species not traditionally considered uropathogens, but may have an indirect relationship to UTI susceptibility at the PUPV site. Multiple species of the microbiome have been implicated as crucial influencers of immune system development especially during the first few years of life. *Bifidobacteria* sp, *Lactobacillus* sp, and *Clostridia* sp have all been linked to inducing development of T regulatory cells in the colon (30–34). *Bifidobacteria* sp abundance has further been shown to enhance the immune response to vaccination, while low species levels correlates with rising allergy and autoimmune diseases (35–37). *Bacteroides fragilis* has been associated with B-cell maturation during the infant period of life (38). In our infant cohort, we saw increases in all 5 of these important species at the PUPV site. Future studies are needed to evaluate whether these species exert any of the same immunological effects in the PUPV region or the urogenital tract, and how that may contribute to local responses to infection and other insults.

Our data regarding changes between individual milestone cohorts showed an evolution driven by developmental milestones, but also revealed some other informative results. For example, despite the close anatomic proximity of the perianal and PUPV environments even in the smallest of patients such as our newborn group, these areas possess their own unique signature. Surprisingly, our premenstrual girls demonstrated overlap between their perianal and PUPV microbiomes even though that cohort should have the greatest anatomic distance. There are multiple possible contributors to the personalization at this site, including but not limited to: variation in age, diet, Tanner stage, hormone levels, personal hygiene habits, and materials of clothing. The importance of a unique environment for each of these sites cannot be over emphasized. A healthy perineal microbiota could impact UTI susceptibility through a variety of mechanisms, including competitive inhibition, maintenance of a pH environment resistant to uropathogens, production of hydrogen peroxide, direct inhibition or bactericidal activity against uropathogens (39–41), and altered antimicrobial peptide expression (42). A pediatric perineum has less separation between the urinary, reproductive, and gastrointestinal tracts which would further support the influence these systems might have over one another. In addition, the youngest of children have poor separation between urine and stool due to being diapered. Perhaps, this anatomic proximity necessitates a separate armamentarium of flora to help maintain genitourinary health given the contamination of overlapping organ systems and similarly explains the shift seen particularly between infants and toddlers as described above.

The second aim of our study compared a group of UTI-prone individuals to aged matched healthy cohorts because while a single UTI alone can be disruptive, an even more challenging clinical scenario occurs when an individual develops repeated infections. A child who suffers from one UTI has a risk of recurrence ranging from 12 to 30% (10); and 25% of adults with a first UTI will have a subsequent infection within the next year (43). Recurrent UTIs result in multiple discrete antibiotic exposures or sometimes the initiation of long-term antibiotic prophylaxis. The impact of antibiotics on the gastrointestinal microbiome has been well-documented: significantly altering that of newborns (44, 45) and even transiently altering the adult gastrointestinal microbiome after a seemingly routine course of therapy (3–10 days) (46–49). While these studies suggest normalization of the gastrointestinal microbiome over time, it may not fully recover until 1-year following antibiotic cessation (50). The consequences of such alterations during the acquisition of one's gastrointestinal microbiome has been linked to multiple disease states (3, 5), but little is known regarding the impact of disruptions in the PUPV microbiome during its maturation. Specific to patients with a history of multiple UTIs, the long-term effects of repeated antibiotic exposure during the establishment of the perineal microbiome in children is unknown. Altered microbiomes following an initial UTI and course of antibiotics could itself lead to increased susceptibility for UTI recurrence. The result could be a cycle of multiple infections in a short time frame, limiting the ability of the microbiome to rebound and further compounding the risk of subsequent UTI. The impact of such a cycle occurring early in development during what we have already demonstrated is a highly dynamic time is uncertain and concerning.

Our data comparing our UTI-prone to UTI-naïve cohort is compelling for the disruption that appears to occur with UTI. A history of UTI significantly changed all microbiomes, but specifically that of the PUPV site. This data is consistent with adult data that shows UTI-prone women have altered vaginal microbiota as compared to healthy controls, marked by diminished *Lactobacillus* sp (41). Our data demonstrates the same findings but in premenstrual children. While our results identify the dysbiosis associated with UTI, they do not define an actual link between such changes in the PUPV microbiome and UTI susceptibility. Whether the shift in microbial composition between healthy girls and those with a history of UTI is a result of their prior infection and/or the antimicrobial exposure remains unclear.

One tempting rationale for defining the normal flora and variations in children who experience UTI is development of a new predictive tool to identify those most at risk for UTI and target early treatment or prevention. By recognizing “protective” flora, we could manage UTIs by offering an antibiotic-independent option augmenting the spontaneously occurring microflora, a particularly appealing solution in this era of practicing responsible antimicrobial stewardship. An unintended observation of this study was the finding that one-third of our UTI cohort (5 subjects) had a documented allergy to an antibiotic, whereas none of the 60 healthy children had a single documented antibiotic allergy. This observation could be a result of bias due simply to exposure differences between the populations; however, it is a reminder that consequences of antibiotic use extend beyond the effects on one's personal microbiome.

Finally, our experience highlights the critical need for pediatric-centered microbiome inquiry. We chose the retroauricular crease based on reports describing the site as stable with a plan of using it as a “control” site. Grice et al. was the first to indicate among a small cohort of 10 adults that among various skin areas, the retroauricular crease site maintained its stability (23). A study using 300 healthy adults from the Human Microbiome Project (HMP) asserted the retroauricular crease was stable for >200 days (51), corroborated by another study which found the area to be unchanging for at least 2 years (24). Prior to collecting our samples, pediatric literature was rare, with one study of 31 infants suggesting the skin microbiome does evolve with age (52). More recently, larger studies published describe the pediatric skin microbiome changes with age and environmental exposures (53). Early life skin microbiota varies across individuals, differing between preterm and full-term infants, between body site, and with developmental age (54). Our data is consistent with these later reports, affirming that even in this previously described “stable” location, age and developmental stage significantly influences its microbiota.

Pilot studies have many inherent limitations in their design which must be considered when interpreting results. Our study examined a small sized sample from a single institution. Our UTI-prone and UTI-naïve cohorts had important differences in racial composition and birth history which could affect our findings. In the adult literature, well-characterized differences in the vaginal microbiomes between women of African-American descent compared to women of European descent have implications in susceptibility to infections like bacterial vaginosis and reproductive outcomes (55–57). We evaluated the impact of race on microbiome diversity in our cohort, finding that race affected the perianal microbiome and not that of other sites, but acknowledge our small sample size prohibits definitive clarity. We were unable to evaluate the impact of birth mode, however, again due to limitations in patient number. It is notable that 40% of the UTI-prone girls were delivered by cesarean section whereas none of UTI-naïve girls were, suggesting that foregoing the passage through the maternal vaginal flora could be a risk factor of UTI, but warrants further investigation. Lastly, we began this project at a time when whole genomic sequencing was not as readily accessible and thus used older 16s rRNA techniques. In general, this study provides compelling preliminary data suggesting an evolution and maturation of the perineal microbiomes and advocates for larger, longitudinal studies evaluating the effects of the PUPV microbiome on UTI susceptibility.

## Conclusions

Significant shifts in the perianal and PUPV microbiome compositions occur during childhood, corresponding with important developmental milestones. There are significant differences in the PUPV microbiome, with even changes in the perianal microbiome, of girls with a history of UTI, likely influenced by both the UTI and the antibiotic exposure. A better understanding of how the perineal microbiomes of healthy children compare to those with a history of UTI could provide a target for UTI prevention. Using the adult paradigm, elucidation of the constituents of the perineal microbiomes in health and disease could provide alternative non-antibiotic approaches to limit UTIs.

## Data Availability Statement

“The datasets presented in this study can be found in online repositories. The names of the repository/repositories and accession number(s) can be found below: NCBI SRA, PRJNA670727”.

## Ethics Statement

The studies involving human participants were reviewed and approved by Institutional Review Board at The Research Institute at Nationwide Children's Hospital. Written informed consent to participate in this study was provided by the participants' legal guardian/next of kin.

## Author Contributions

EL, CC, and SJ conceived of the project, designed the study, and secured funding and reviewed all raw data, synthesized into appropriate analyses, and wrote the manuscript. SS, SD, and PK assisted with the technical aspects of the study design, analyzed the microbiota data, and provided the initial analyses. EL and CC recruited all patients and obtained all samples. PK made minor contributions to the manuscript. All authors contributed to the article and approved the submitted version.

## Conflict of Interest

The authors declare that the research was conducted in the absence of any commercial or financial relationships that could be construed as a potential conflict of interest.

## Supplementary Material

Supplementary Figure 1 Comparison of the microbiomes by race in the healthy cohorts and UTI-prone girls. **(A)** Unweighted UniFrac profile evaluating for differences in community members, **(B)** weighted UniFrac profile of community structure, and **(C)** the Bray-Curtis dissimilarity index determined for the saliva, retroauricular, perianal, and periurethral/perivaginal sites. Each data point represents the bacterial community of a given subject in Caucasians (green), African Americans (AA; red), and Others (purple). There was a significant difference between races in the weighted and Bray-Curtis analysis of the perianal microbiome. Color clouds are intended for ease of visualization and do not provide any statistical value. Statistical significance was determined by ANISOM analyses.

Supplementary Figure 2 Complete list of bacterial species on Deseq that were more highly expressed in UTI-prone vs. UTI-naïve groups (in blue) as compared to bacterial species that are expressed less in UTI-prone vs. UTI-naïve groups (in orange), reported as log^2^ fold change.

## Abbreviations

DNA: deoxyribonucleic acid
dsDNA: double stranded deoxyribonucleic acid
EMR: electronic medical record
LDA: linear discriminant analysis
OUT: operational taxonomic unit
PCoA: principle component analysis
PCR: polymerase chain reaction
QIIME: Quantitative Insights into Microbial Ecology
rRNA: ribosomal ribonucleic acid
UTI: urinary tract infection.
