# Supplementary figures and images for: Acquisition, Divergence, and Personalization of the Female Perineal Microbiomes Are Driven by Developmental Milestones and Disrupted by Urinary Tract Infection: A Pilot Study
Source: Front Pediatr. 2020 Dec 8;8:542413. doi: 10.3389/fped.2020.542413 (PMC7752998; doi:10.3389/fped.2020.542413)

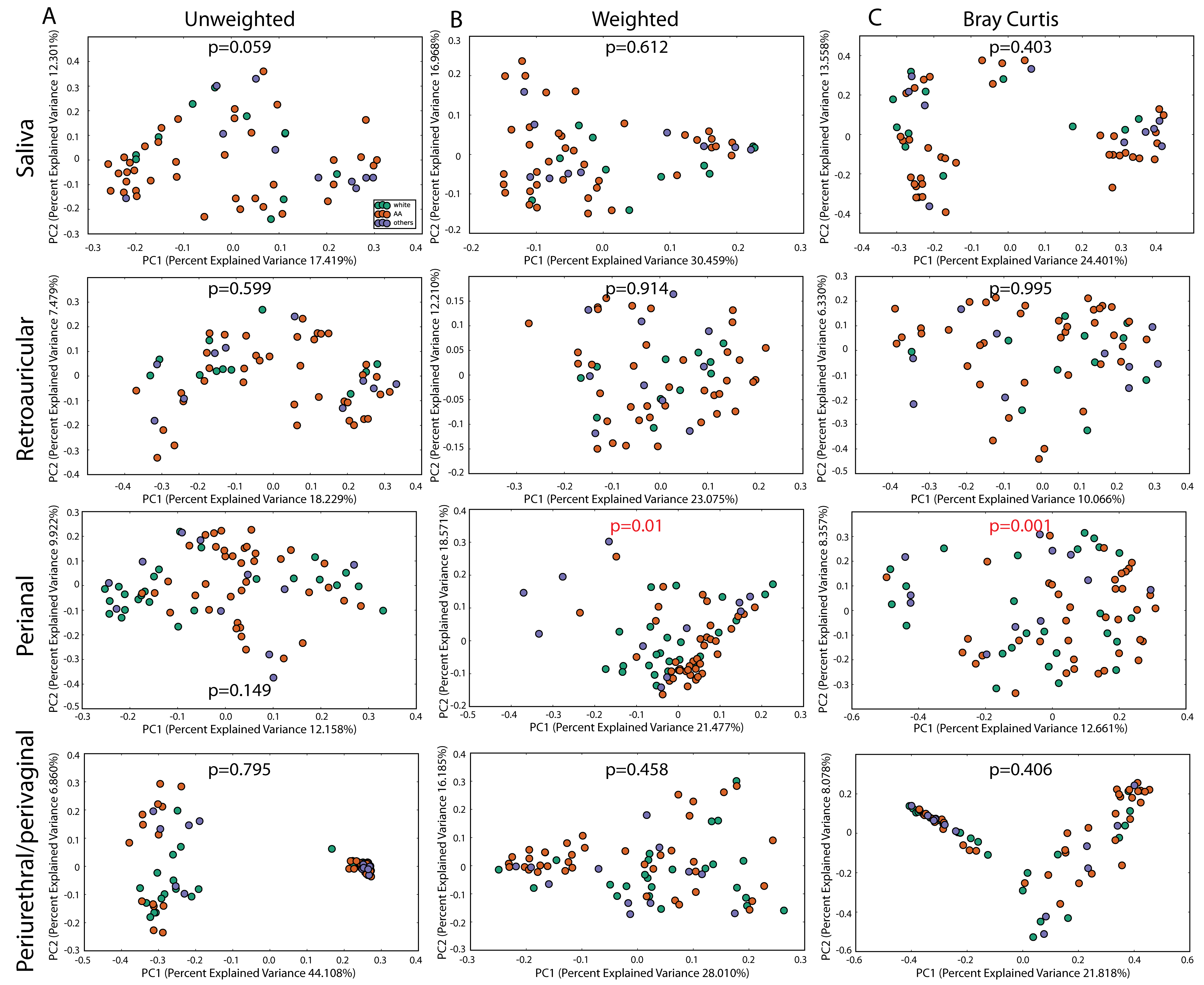

Supplement: Supplementary Figure 1 — Comparison of the microbiomes by race in the healthy cohorts and UTI-prone girls. (A) Unweighted UniFrac profile evaluating for differences in community members, (B) weighted UniFrac profile of community structure, and (C) the Bray-Curtis dissimilarity index determined for the saliva, retroauricular, perianal, and periurethral/perivaginal sites. Each data point represents the bacterial community of a given subject in Caucasians (green), African Americans (AA; red), and Others (purple). There was a significant difference between races in the weighted and Bray-Curtis analysis of the perianal microbiome. Color clouds are intended for ease of visualization and do not provide any statistical value. Statistical significance was determined by ANISOM analyses. [file Image_1.tif]

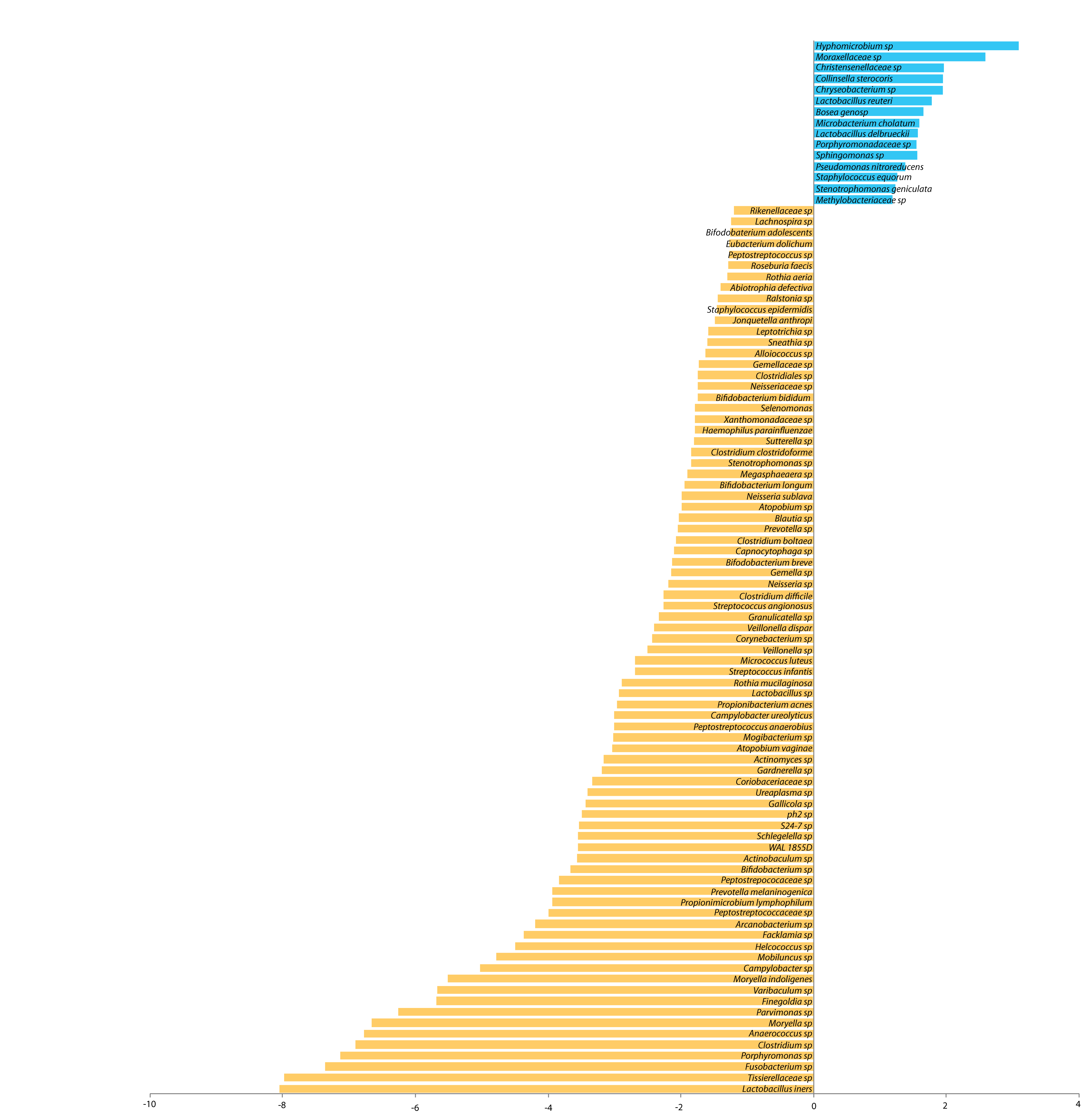

Supplement: Supplementary Figure 2 — Complete list of bacterial species on Deseq that were more highly expressed in UTI-prone vs. UTI-naïve groups (in blue) as compared to bacterial species that are expressed less in UTI-prone vs. UTI-naïve groups (in orange), reported as log2 fold change. [file Image_2.tif]
